# Five pillars for stakeholder analyses in sustainability transformations: The global case of phosphorus

**DOI:** 10.1016/j.envsci.2020.02.019

**Published:** 2020-05

**Authors:** Christopher Lyon, Dana Cordell, Brent Jacobs, Julia Martin-Ortega, Rachel Marshall, Miller Alonso Camargo-Valero, Erin Sherry

**Affiliations:** a Sustainability Research Institute, School of Earth and Environment, University of Leeds, Leeds LS2 9JT UK; b Institute for Sustainable Futures, University of Technology Sydney, Broadway NSW, 2007, Australia; c Lancaster Environment Centre, Lancaster University, Lancaster, LA1 4YQ UK; d BioResource Systems Research Group, School of Civil Engineering, University of Leeds, Leeds LS2 9JT, UK; e Agri-Food and Biosciences Institute, Belfast, BT9 5PQ, UK

**Keywords:** Phosphorus, Sustainability, Transformations, Stakeholders, Natural resources, Food systems, Food security, Agriculture, Water

## Abstract

Phosphorus is a critical agricultural nutrient and a major pollutant in waterbodies due to inefficient use. In the form of rock phosphate it is a finite global commodity vulnerable to price shocks and sourcing challenges. Transforming toward sustainable phosphorus management involves local to global stakeholders. Conventional readings of stakeholders may not reflect system complexity leaving it difficult to see stakeholder roles in transformations. We attempt to remedy this issue with a novel stakeholder analysis method based on five qualitative pillars: stakeholder agency, system roles, power and influence, alignment to the problem, and transformational potential. We argue that our approach suits case studies of individual stakeholders, stakeholder groups, and organisations with relationships to sustainability challenges.

## Highlights

• Stakeholders hold complex roles, capacities, power.
• Stakeholders can be read as complex entities within complex social-ecological systems.
• Deeper reading of stakeholders is important for identifying leveragers for sustainability transformations.

## 1 Introduction

Human activity is hitting the boundaries of a stable Earth life-support system. To achieve sustainability, we need to fundamentally restructure our activity on the planet (Blythe et al., 2018). To achieve such fundamental change, human systems need to *transform* in the sense that the underlying *intent* drives a more sustainable trajectory (Abson et al., 2017). Sustainability transformations occur through individual and collective action, social structures and institutions. Stakeholders, those individuals and groups (agents or actors) “who can affect or are affected by” (Freeman, 2010, p. 49) a given sustainability problem, are central to such transformations.^1^ Understanding stakeholders as key actors means capturing peculiarities beyond system complexity. These include the influence of abstract thinking, forms of agency, and institutions that differentiate us from non-humans and make us innovative, obstinate, prone to conflict, accommodating, and cooperative in the face of complex social-ecological challenges (Adger, 2000; Brown and Westaway, 2011; Chandler and Reid, 2016; Davidson, 2010; Ostrom, 2000). Accommodating these traits as part of the transformation process requires careful appreciation of the uniqueness of individual people and organisations in the personal, institutional, and societal contexts of many present environmental challenges (Fazey et al., 2018; Gram-Hanssen, 2019; O’Brien, 2018, 2016). Researchers are therefore challenged to create methodological innovations for sustainability transformations that centre on greater stakeholder involvement to drive sustainability change processes across the range of societal and personal spheres of action (Fazey et al., 2018b; O’Brien, 2018; Steffen et al., 2015; van der Hel (2018); Wiek and Lang, 2016).

Centring stakeholder involvement thus requires involving a rich understanding of who those stakeholders are, what role can they play in transformation, and how can it be enabled (Jacobs et al., 2017). Efforts to date tend to focus on the various means of defining, identifying, measuring, categorising, and engaging the optimal array of stakeholders for a given sustainability problem, including less obvious stakeholders and those who would be otherwise marginalised (Colvin et al., 2016, 2015; Cordell et al., 2015; Graham and Ernstson, 2012; Rastogi et al., 2010; Reed et al., 2009). Yet researchers continue to identify shortcomings in how stakeholders are understood and selected (Colvin et al., 2016). The criticism of stakeholder analysis persists because whether an approach accommodates direct or peripheral stakeholders, or uses one definition of stakeholder or another, research may not apply a systematic means of seeing stakeholders as dynamic entities in their own right. Further, these shortfalls may permit a lack of close attention to the pitfalls of the actor-driven power and politics of sustainability transformation (Blythe et al., 2018). Capturing only obvious stakeholders based on their clear instrumental role in a specific issue presents a fundamental problem in that transformational stakeholders can be overlooked. Missed stakeholders may be the leveragers who lever the leverage points within a system, tipping it toward transformation.

To overcome the challenge described above, we propose a transformation-geared approach to stakeholder analysis inspired by complex social-ecological systems thinking that understands stakeholders as dynamic actors with multiple, mutable, and sometimes enmeshed roles, values, and capacities within a system facing an environmental problem. We categorise and assess stakeholders using an innovative approach based in part on the construction of avatars^2^ commonly used in complex role play games. Doing this allows us to identify stakeholders along multiple axes that account for their power and influence, support for or orientation toward a sustainability issue, temporal and spatial locations, and other dynamic attributes. Our novel approach combines five pillars (agency, system role, power and influence, alignment, and transformational readiness) and avatar construction (Schrader, 2019) to categorise and assess stakeholders. We test this approach using the pressing global food and water sustainability challenge of phosphorus (chemical element symbol ‘P’) (Li et al., 2019; Steffen et al., 2015). Phosphorus is an element essential to agricultural fertiliser and therefore food security, yet is used very inefficiently, remains vulnerable to supply disruption, and contributes to severe water pollution (Cordell et al., 2015; Reitzel et al., 2019; Withers et al., 2019). Importantly, recent research also argues that phosphorus-specific sustainability transformations require close attention to a wide range of system stakeholders outside the direct or linear producer-consumer supply chains to include those who can leverage change (Withers et al., 2019).

Our paper is organised as follows. First, we briefly describe the phosphorus sustainability challenge. Next, we frame our argument in reference to the sustainability stakeholder literature to show how previous approaches are essentially limited in their capacity to enable substantial transformation. Following this, we introduce our five-pillars approach to stakeholder categorisation and critical analysis that aligns with a view of the world as a complex social-ecological system, which we exemplify with the P example. We conclude with a summary and final remarks regarding the development and application of our approach.

## 2 The phosphorus sustainability challenge

The global food system is both a driver of, and subject to, anthropogenic environmental change processes. Recent research has stressed the vulnerability of this system to shocks and stresses due to the assemblage of climate change impacts, diets, biodiversity losses, agricultural and land-use practices, and resource depletion (Cottrell et al., 2019; Myers et al., 2017). Underpinning the food system is the relative availability of the key plant nutrients necessary for crops and livestock feed, and upon which all life depends. From the various key plant nutrients, phosphorus represents a critical planetary boundary and sustainability challenge (Cordell, 2010; Steffen et al., 2015). All major agricultural systems today are dependent on fertilisers derived from finite phosphate rock, where global supply is unevenly distributed and even export-restricted among major and minor producers (e.g. Morocco and China) creating uncertainty over continued access (Blackwell et al., 2019). This means that projected supply disruptions or price spikes in coming decades will likely have severe consequences for any country’s current agri-food systems (Cordell and Neset, 2014). The scarcity and geopolitical risks associated with this critical resource, coupled with widespread phosphorus pollution of water, and a serious ambiguity around stakeholder roles and responsibilities, creates a truly wicked sustainability challenge for which governance requires urgent attention (Withers et al., 2019). Agricultural phosphorus is provided in mineral form from phosphate rock, and in organic form from livestock manure and slurry, sewage biosolids, and compost. Mineral (rock-derived) phosphorus when combined with nitrogen and potassium as “NPK” is the most commonly used agricultural fertiliser in global agricultural production systems. Multiple issues, such as price increases or other [potential] supply disruptions such as trade disruptions (e.g. the UK’s Brexit), the impact of phosphorus on water systems (e.g. eutrophication), poor understanding of phosphorus dynamics in soils, losses between the farm gate and dinner plate (food waste management), and lack of up-scaled recovery technologies, result in a highly inefficient and import-dependent phosphorus system reliant on a single source (Cordell and Neset, 2014; Cordell and White, 2014, 2011; Withers et al., 2019). Disruptions to this system can have profound impacts on agricultural economies, national or global food security, and ecological integrity.

Scientists have identified the critical need to move global phosphorus use to a much more sustainable footing (Li et al., 2019; MacDonald et al., 2011; Steffen et al., 2015). Institutions, however, have yet to define and project a clear *intent* towards improving the sustainability of phosphorus within food systems. At the time of writing, no enforceable overarching national or global governance or management structure exists for phosphorus use anywhere in the world, although there are research and stakeholder platforms in a number of regions. For example, the European Sustainable Phosphorus Platform^3^ is one of a handful of national or regional nutrient platforms, mostly within the global North, that bring together a range of industry, government, and research stakeholders for conferences, workshops, and other knowledge sharing events. Some phosphorus platform members may use practical and experimental technologies for improving phosphorus use in agriculture through precision farming techniques, and recovering phosphorus from wastewater or points on the food system (Cordell et al., 2011; Mihelcic et al., 2011). However, these platforms and technologies are not yet incorporated at scale, or within a formal strategic programme of phosphorus or food system sustainability. A key step in developing such initiatives involves not just identifying the key stakeholders, but also those who might leverage the kind of systemic change needed to shift phosphorus use to improve food and water resources (Jacobs et al., 2017). At a basic level, phosphorus system stakeholders include farmers, wastewater companies, government regulatory agencies, policymakers, fertiliser companies, and scientists.^4^ Considering global sustainability is ultimately delivered *via* national policy and research agendas (Bhunnoo and Poppy, 2020), these stakeholders are likely to exert influence over phosphorus sustainability by shaping both the intention and design of national food system transformations. This added complexity means it is even more important to consider stakeholders within a multi-faceted and dynamic framework.

## 3 Stakeholder analysis in sustainability transformations

Sustainability research tends to take a one-dimensional interest or role-based approach to exploring different ways of recruiting or describing stakeholders (Clegg and Pitsis, 2012; Colvin et al., 2016; Fazey et al., 2017; Redpath et al., 2013; Reed et al., 2009; Tompkins et al., 2008). For example, to study phosphorus sustainability within the North American food system, researchers conducted workshops with the board of directors and other stakeholders from academic, biotechnology, fertiliser makers, and agricultural organisations involved with the Sustainable Phosphorus Alliance (SPA), 2020 (Jacobs et al., 2017).^5^ Despite the advocacy of the SPA as an expression of stakeholder agency, the selection of the stakeholders themselves was based on their involvement with the SPA and their knowledge of the current system and not a closer assessment of what might be unique about each stakeholder beyond an objective role (e.g. as academics or fertiliser companies), or potential future role in a transformed system that may also need stakeholders which currently do not exist. In an example of an extensively farmed catchment, this lack of a detailed understanding of the system and how its stakeholders (farmers) were reacting to catchment specific issues, saw the phosphorus content of a lake rise significantly over a multi-year period (Schulte et al., 2009), leading to questions about the relative representation, legitimacy, and accountability of stakeholders that closer attention would help to answer.

This type of stakeholder understanding may overlook the particulars of agency that a stakeholder holds, and miss capturing the inner “personal sphere” of transformation that covers complex moral positionings, worldviews, and other unique axiological attributes that individual stakeholders embody (Ives et al., 2019; O’Brien, 2018). Indeed, a diversity of stakeholders has been modelled to correspond to a strong positive relationship to social-ecological systems resilience in the face of sustainability challenges (Grêt-Regamey et al., 2019).

However, the unique qualities and dynamics of each stakeholder are where the mechanisms for transformational action rest: stakeholders must be doing something or be willing to do something for sustainability objectives, deliberately or not for transformation to be possible. Moreover, whether and under what conditions a given stakeholder subscribes to and acts toward sustainability may reflect their moral or ethical orientation in addition to their given interest or literacy in the issue (Cohen et al., 2015; Mathur et al., 2008). Therefore, important dimensions to understanding stakeholders are the ethics and morals of what stakeholders value in terms of their relational responsibility in descriptive, normative, and instrumental dimensions to a sustainability problem (Mathur et al., 2008).

Some work is now beginning to capture more nuanced readings of stakeholders, again through participatory workshop processes. Pereira et al. (2018), for example, in seeking stakeholder diversity for participatory future visioning workshops, recruited diverse “key thinkers, artists, scientists, and change makers” from different age, gender, and cultural backgrounds who were also recognised as “social innovators who have agency in and influence in an array of social networks and institutions”. However, stakeholder participants in this work were identified through snowballing from existing author networks. This common selection method suggests, that despite more careful attention to qualities of individual stakeholders, unless researchers (or their equivalents) are particularly well-networked with the appropriate communities or gatekeepers, identifying and accessing more specific stakeholders would likely remain a frustrating disadvantage to anyone without a similar level of power and influence to access these groups. In this way, researchers themselves are stakeholders with a degree of power and influence.

Building on the trajectory of existing stakeholder recruitment and assessment, particularly for visioning or creating more positive sustainable futures (transformations) we propose a more methodical transformations-geared approach to assessing stakeholders. We seek to capture their structural and agential qualities within a system to help identify those who fit different methodological roles or research goals. By providing a means for a richer reading of stakeholders without always interviewing them, our proposed approach may also help to overcome problems of access for less well-networked researchers or stakeholders, and provide a means of understanding, through secondary sources, those stakeholders whom researchers may not be able to engage.

## 4 A new approach to understanding stakeholder for transformations

Social-ecological systems (SES) are conceptualised as comprising dynamic human and natural entities that interact in complex ways at, and across, different rates and scales in space and time (Welsh, 2014). In addition to being influenced by or influencing (Freeman, 2010) a social-ecological problem, stakeholders can be viewed as individuals and representatives of groups or domains of values, beliefs, norms, and capacities that also crosscut each other, some of whom may be more innovative or amenable to change than others. Stakeholder domains differ according to the specific sustainability challenges under consideration. Our approach is anchored in five pillars that together reflect different expressions of agency, roles (current and potential) and influence of stakeholders within a system, which together help reveal crosscutting issues of representation, legitimacy, and accountability.

1 An understanding of stakeholders that includes agency;
2 An identification of stakeholders in their systems roles and dynamics;
3 Power analysis;
4 Identification of interest, moral orientation, and alignment with sustainability;
5 Stakeholder transformational readiness.

In this section, we describe the pillars of our approach, supported by a selection of the relevant published literature illustrated with examples relevant to the P global challenge.

### 4.1 Pillar 1: agency

In SES, and in contrast to non-human systems, human systems produce institutions such as laws, governments, cultures, and markets that nonhuman systems do not have. Humans also have agency, meaning they can individually or collectively interpret, reflect upon, and abstract their reality in ways that shape and condition their actions, sustainable or otherwise (Brown and Westaway, 2011; Davidson, 2012, 2010; Lyon and Parkins, 2013). Agency in stakeholder complexity can translate into the various motivations for participating in knowledge production that may not complement research priorities and requires a deeper understanding of stakeholder values, needs, and motivations (Lavery, 2018). We therefore understand stakeholders to be “specific real-world [human] groups, organisations or significant individuals” (Cordell, 2010, p. 43).^6^

However, we also take inspiration for our approach from Karen Barad, who speaks of “agencies” as the doing of agents or actors (Barad, 2010, 2007). Agents ‘intra-act’ and are constituted by and also constitute the relationships between events, discourses, and material entities, which means that they cannot be understood as discreet or independent entities in their own right. Important to our argument, Barad (2010, 265) speaks of the “irreducible heterogeneity” of the assemblage of agents, meaning that their relationships with each other, the internal function of their organisations, the material, spatial, and temporal world cannot be disaggregated. As an agent, a stakeholder is therefore more than just their role in an organisation or contribution to a problem, which is what often defines their inclusion, exclusion, and analytical strategy in stakeholder or key-informant based studies. While the focus on their role in an issue is clearly important, considering stakeholder agency as an expression of relationality accounts for the heterogeneity of who and what they are in terms material, functional, ethical, moral, existing and potential capacities, and other categorisations. Stakeholder comments, bearing, and actions can thus be viewed as the manifestation of complex relational assemblages that, in Barad’s terms, are entangled with events occurring at different locations in space and time (Barad, 2010).

Stakeholder analyses generally see values, culture, knowledge and other attributes unique to humans as expressions of agency.^7^ Thus, regarding stakeholders as agencies, not just agents, pluralises them and more aptly captures the verb form of the noun *agent*. This rhetorical act turns stakeholders into actors who are acting and becoming. This makes them dynamic entities (complex adaptive systems in their own right), and not just machine parts performing defined and fixed functions or holding interest in a system or sustainability problem. Inspired by Barad as a heuristic fillip, the remaining pillars categorise stakeholders to capture their complex expressions of their agency. Agents also have potential, a key element of both social-ecological systems (Leslie et al., 2015), and of Barad’s analysis. Recognising a given agent’s potential also helps us to understand the roles stakeholders do or do not, could or could not play. This can be accomplished by considering the dynamic interactive effects on agents across the pillars. For example, a start-up with new phosphorus recovery technology could potentially play a much more significant role in phosphorus sustainability than they currently do, with the support of a favourable enabling environment (an increase in power leading to a larger role in the system). On the other hand, a major chemical fertiliser producer relying on Moroccan phosphate rock may not have a major role in the transformed system of the future, but could use its present set of legal and market resources (power and influence) to limit the role of new players in the fertiliser market who might challenge its business model (protecting its interest). Two farmers, who are agents with a similar role in the system, could, due to diverse outlooks, knowledge, or access to resources, react very differently in the face of a sustained increase in fertiliser prices, with one maintaining the status quo and the other, seeking novel/innovative ways to apply fertilisers more efficiently. But what larger role do such agents play and where are the trade-offs?

### 4.2 Pillar 2: system roles

A next step in categorising stakeholders from a complex system perspective is to understand their conceptual role (or potential role) within the (transformed) system. In addition to their objective ‘real-world’ role, such as government ministry or water service provider, stakeholders perform specific functions or influences on the system. Drawing on Cordell’s (2010) adaptation of Soft Systems Methodology (SSM) (Checkland and Poulter, 2006), we further adapt the SSM model to identify a series of key system stakeholder roles: regulator, decision-maker, guardian, owner, advocate, catalyser/blocker, winner/loser (or beneficiary/disadvantaged), and seller/buyer. Table 1 consolidates and defines this list of these system actor roles. A *regulator,* for example makes the rules and sets the standard by which the system operates, e.g. a government environment agency. A *decision-maker* sets rules, courses of action, and other processes.

**Table 1:** Different stakeholder roles within a complex system (adapted and expanded from Checkland and Poulter, 2006) with examples relevant to the phosphorus-food challenge.

System Role	Definitions	Phosphorus examples
Regulator	Makes the rules and sets the standard by which phosphorus is used and managed.	Lawmakers, government agencies, etc. making and enforcing water and landscape regulations, and trade agreements for phosphorus.
Decision-maker	Holds a key visible or hidden, formal or informal decision-making ability that influences different parts of the system.	Farmers deciding on fertiliser type and application, or a water company deciding on phosphorus recovery technology.
Guardian	Performs the monitoring, evaluation, and enforcement functions within the system to maintain system functioning with sufficient authority and trustworthiness.	Non-partisan environment and food standards agencies, international trade-regulators.
Owner	Perform control functions in the system but may not be a direct decision-maker or guardian.	Phosphorus fertiliser producers, farmers, or water managers.
Advocate	Promotes certain functions or potential functions in the system,	A sustainable phosphorus platform or environmental organisation.
Catalyser/Blocker	Catalyser instigates or stimulates courses of action within the system, while a blocker inhibits.	A sustainability-promoting organisation that that can mobilise key phosphorus stakeholders, or an industry group that may lobby against sustainable phosphorus management (as with oil companies and climate change).
Winner/Loser	Winners benefit from current or changing/changed system; while a loser is disadvantaged if it does not adaptively transform.	Current winners are mining operators in Morocco, and losers are water users in the UK, and vice versa.
Seller/ Buyer	Financial or market-based exchange relationships within the value chain of the system.	Buyers and sellers of different forms of phosphorus or technology.

Any complex system must have the capacity to self-organise, hence contain elements of monitoring, feedback and control. These functions are overseen by a system’s *guardian*. Surprisingly, despite the critical role of phosphorus underpinning all food and water systems, there is often no clear guardian, although some existing agents have the potential to fill this role, such as the UN’s Food and Agriculture Organisation, or a national Department of Agriculture, responsible for monitoring and ensuring phosphorus availability for food production. Thus, transforming to a sustainable system could explicitly envisage and (re-)define the roles and responsibilities for such an actor (Cordell, 2008). We stress that in our formulation stakeholders are complex entities that can hold more than one role, or indeed conflicting roles in relation to a problem. Recognising and explaining these is one way of revealing trade-offs and other differentials around legitimacy and accountability. For example, a supermarket chain with strong national or own-brand P-sustainability practices (e.g. through collaboration with farmers), may nonetheless also host brands or use global supply chains without such elements, thus acting as owner, catalyser, and blocker simultaneously

This complex systems perspective first requires explicit critique of the system boundary, that is, who/what is included, excluded, marginalised and why (Midgley, 2003). Boundaries can be geographical (catchment, regional, global), conceptual (food system, eco-system), political (local government, national), or another defined system. Drawing boundaries has strong implications for which stakeholders are identified as relevant to the transformation, hence included in (or excluded from) the study and ultimately the nature of the study’s outcomes. Young (2002, p. 12) stresses from a resource regime perspective: “a regime that ignores what turn out to be significant elements of an ecosystem cannot produce sustainable results”. Drawing boundaries is inherently value-based, for both the researched and researcher, hence the need to critically explicitly reflect on assumptions (and the values that give rise to them). Boundary critique as a methodological technique can equally be used to interrogate the sustainability of existing system boundaries that may have implicitly or arbitrarily been drawn, or cater to different contexts.

For example, in the context of the global phosphorus system, hotly contested boundary stakeholders are the political authorities that define and control Western Sahara^8^ . From an ethical and corporate social responsibility position, this region is a critical stakeholder, as food consumers and companies are knowingly or unknowingly supporting an occupation that breaches human rights, through the trade and consumption of foods that have been grown using fertilisers sourced from the contested region (Corell, 2002; WSRW, 2017). Further, from a geopolitical food security position, the potential consequences of a disruption of phosphate rock supply from the world’s largest phosphate producing region could be catastrophic for the many national food systems that are dependent on these phosphate rock imports, from the EU to Malawi, and those reliant on importing surplus food from those systems. In another example of boundary critique, until very recently, global environmental change and food systems implicitly excluded phosphorus as a scarce resource or excluded phosphorus altogether (Chowdhury et al., 2017). This meant causal links between phosphate scarcity and food security could not be easily drawn, thus making it more difficult to assess policy implications, stakeholders, or funding of further research on the problem (Cordell, 2008). While recognition of the phosphorus boundary problem is increasing (e.g. Willett et al., 2019), there remains no concerted public international effort to [sustainably] govern phosphorus.

The roles described in Table 1 bear some similarities to those drawn from soft systems methodology used in organisational studies. In a recent review of stakeholder collaboration, Goodman et al. (2017) note the minimal research in sustainability innovation in business and management studies. From interviews with a range of organisations, they identified eight roles played by stakeholders from the civil society organisations, business, academia, and public sectors as: stimulator, initiator, broker, mediator, concept refiner, legitimator, educator, context enabler, and impact extender (Table 2). These are further potential legitimate roles that stakeholders in a transformed (or transforming) system can play. In Table 2, we identify examples for a phosphorus system.

**Table 2:** Stakeholder classifications from Goodman et al. (2017), phosphorus examples by authors.

Organisational role	Definitions^a^	Phosphorus examples
Stimulator	Stakeholder role involving a call for ideas or offer of initial funding to resolve a social or environmental issue that sets the innovation in motion.	Research council or impact investor issuing funding for research into sustainable phosphorus.
Initiator	Initiating, inspiring and/or generating the idea for the innovation. A stakeholder assuming the initiator role may also be actively involved at later stages of the innovation process.	Inventor of a novel phosphorus recovery technology or deeper understanding of phosphorus-soil dynamics.
Broker/mediator	Integrating other stakeholders; Organizing testing, pilots and trials, and collecting feedback.	Entrepreneurs and phosphorus stakeholder platforms.
Concept refiner	Give feedback and technical expertise to make the product/service more attractive to a wider range of end users.”	Farmers, water companies, householders.
Legitimator	Assuring and promoting the brand.	A UN or official non-partisan science organisation backing a sustainable phosphorus initiative.
Educator	Providing, educating and communications.	A public agriculture department providing new phosphorus guidance for farmers.
Context enabler	Dealing with infrastructure and regulation.	Governments or water companies responsible for providing regulation and infrastructure for phosphorus management.
Impact extender	Extending and increasing usage and impact.	A grassroots organisation or NGO taking-up government or scientific knowledge about phosphorus management.

a Direct quotes from Goodman et al. (2017).

Stakeholders may vary in degrees of proactiveness and reactiveness, which *en masse* create a complex set of interactions that would promote or detract from sustainability agendas. While the efficacy and meaning of sustainability for each stakeholder were not assessed in Goodman’s study, their framework does classify stakeholders by role consistent with their complex interactions across social sectors. Checkland and Poulter (2006) and Goodman et al. (2017) also provide alternate or complementary social classifications. Though power is implicit in these roles, the specifics of power are essential enough to form our third pillar.

### 4.3 Pillar 3: power

A power analysis helps us understand the relationship between the stakeholder and the system in terms of how their relative influence manifests in practices (or may manifest in future practices). Does a stakeholder have strong legislative power over the environment? Does the stakeholder manage a large river catchment, or are they a local grassroots organisation with informal sway, such as a farmer or fertiliser producer? Answering these kinds of questions permits an assessment of where the current and potential power rests in the system – a noted tricky gap in multi-stakeholder (polycentric) approaches to environmental governance (Morrison et al., 2019).

A number of researchers have consequently proposed ways of understanding the forms of power stakeholders wield in sustainability problems (Avelino and Rotmans, 2011; Braunholtz-Speight, 2015; Brisbois and de Loë, 2016a; May, 2015). For water management, Brisbois and de Loë (2016a, 2016b) draw on Lukes (2005) to identify instrumental, structural, and discursive dimensions of power. Instrumental power is causal power that is “characterized by overt competition for influence and measurable use of resources in that competition” by those with the means to do so (Brisbois and de Loë, 2016b). Structural power is decision-making and agenda-setting power permitting inclusion or exclusion of certain groups, ideas, or practices. Discursive power refers to manipulation of an actor by another, which may include covertly shaping their interests. May (2015) draws on others (Bourdieu, 1986; Foucault, 1980; Mann, 1993) to discuss concepts of structural, differential, and embedded power in the context of fisheries management. Here, structural power refers to regulations, institutions, bureaucracies, and organisational processes that shape resource management processes. Differential power is the relative power of competing social groups or institutions. May’s embedded power refers to the norms, values and preferences that shape social interactions.

Another Lukes (2005) derived example is Gaventa’s powerplane (Gaventa, 2006) which places power across three dimensions (visible, hidden, and invisible) and several levels (individual, community, etc.) operating via closed, claimed and created spaces. Visible power, as an instrumental power, refers to the power of clear rules, norms and sanctions that govern behaviour. Hidden power is the structural power of empowered decision-makers to set rules and norms that shape social structures. Invisible power (similar to May’s (2015) embedded notion) is the power of ideas, habits, and culture “that people may unconsciously act through” (Braunholtz-Speight, 2015, p. 126; Gaventa, 2006). Closed, claimed, and created spaces are read as locations in a power structure that are inaccessible to some, such as political decision-making by marginalised groups, or cabinet deliberations inside formal governments. Claimed spaces refer to how the stakeholder is able to claim representation within a decision-making structure. Created spaces are those spaces that a group may manifest to provide them with power, for example by setting up a new organisation able to advocate or collect resources.

A third view identifies innovative, constitutive, and transformative forms of power that can operate through antagonistic and synergistic dynamics (Avelino and Rotmans, 2011). Innovative power refers to the “capacity of actors to create or discover new resources” (Avelino and Rotmans, 2011, p. 799). Constitutive power is the way actors “constitute the distribution of resources, by establishing, enforcing and reproducing existing structures *(formal/informal laws, norms, paradigms, traditions*) and institutions (*organizational and physical infrastructures*)” (Avelino and Rotmans, 2011, p. 799). Transformative power is the ability to change the forms, ways and means by which resources are distributed. Avelino and Rotmans (2011) also attempt to capture the dynamic qualities of power. Their antagonistic power refers to the power to resist or restrict other types of power; and, synergistic power occurs where groups enable and empower each other (Avelino and Rotmans, 2011, p. 799).

We considered these discussions of power and compared them to our combined experience in sustainability studies and the problem of phosphorus sustainability (Cordell, 2010, 2008) to produce a provisional list capturing stakeholder roles and dynamics, and expanding the list to include some of our own types (Table 3). We add *economic power* which we define as purchasing power (demand), the economies of scale to supply a good or service, or the relative market or trade power of a given economic actor. *Latent or potential power* in turn refers to actors identified during the research with potential to be more influential under future conditions. We further define *ambivalent and blackbox power* to describe forms of power held by actors whose views or actions remain opaque until they act, perhaps at the last minute, but will influence systems for better or worse. For example, the swing voters on an institutional board or general election can decide outcomes: UK pro-Brexit voters were not expected to win, yet the results of this referendum are likely to have a profound impact on UK food and agriculture (Lang et al., 2017). Lastly, we add the dynamic power qualities of *antagonistic* and *synergistic power* (Avelino and Rotmans, 2011) and we include a third quality of *accommodating power*. Accommodating power describes the middle ground between antagonism and synergy that is the facilitated co-existence of groups through negotiation and comprise. A full list of our stakeholder power categories is given in Table 3.

**Table 3:** Stakeholder power categories, including phosphorus examples.

Power type	Description	Phosphorus examples
Economic or market power	Purchasing power (demand) or economies of scale to supply a good or service; market power (potential for collusion or cartel) and trade power (subject to few tariff and non-tariff barriers)	Farmer fertiliser purchase power; China’s market power as a key global phosphate producer (evidenced in 2008 when China imposed 135 % export tariff contributed to price spike and farmer riots); Trade power with institutional backing to allow for quick adjustments to national food and fertiliser trade flows, including establishment of strategic P reserve; The scale of economic power can be shifted from oligopoly of global producers to localised phosphorus sources (E.g. In the fictitious dystopian future of URINETOWN, all toilets have been privatised due to repeated drought, urinating in public is punished by gross penalties, and the sanitation company own your pee (Kotis and Hollman, 2001))
Knowledge power	Production, exchange, brokering, translation of knowledge	Fertiliser marketing consultancies (e.g. CRU) that produce/sell important market data, kept behind an expensive paywall.
Latent or potential power	Potential power to influence future events, systems or scenarios but not presently	Wastewater treatment plants can be described as ‘sitting on a gold mine’ due to the phosphorus content of wastewater, which may become expensive in the future as it becomes scarcer. Large consumers of ‘clean green’ energy from local bioenergy, can potentially drive/stimulate phosphorus recovery in the future.
Weltanschauung^a^ power	Inherent power associated with different worldviews	Improving economic productivity (e.g. phosphorus efficiency) is a more dominant paradigm than say the right to food or changing diets.
Post-human power	Power of non-human objects or forces	Phosphate rock is a much more ‘powerful’ phosphorus entity than say phosphorus sourced from human excreta.
Persuasive power	People/groups that may hold little practical power but manage to influence situations in a positive or negative direction through persuasiveness.	Some members of the phosphorus research community who are very eloquent and persuasive.
Convening power	Persons or groups who due to their position/status can bring together key people/groups for action	Instigators of regional or national phosphorus platform.
Antagonistic power dynamic	“when one type of power resists or prevents another”^b^	Some fertiliser companies that are resistant to change/innovation and seek to maintain status quo.
Synergistic power dynamic	“when different types of power mutually enforce and enable each other”^c^	Entrepreneurs co-recovering bioenergy from digestion of organic wastes for sale, which also facilitates the co-recovery of phosphorus by-products.
Accommodating d power (dynamic)	not synergistic / mutually reinforcing, but able to coexist through negotiation	Parliaments that set food, agriculture, trade or other policy through deliberative legislative means.
Ambivalent & ‘blackbox’ power	Power held by an actor that is unknown or unforeseeable until it appears in given moment (hence black box)	UK pro-Brexit voters were not expected to win, but did, the results of which are likely to have a profound impact on UK food and agriculture.

a German for ‘worldview’, from Soft Systems Methodology (Checkland and Poulter, 2006).

b Avelino and Rotmans (2011).

c Avelino and Rotmans (2011).

d Intention of ‘seeking system accommodations’ from Midgley’s (2003) Critical Systems Thinking.

### 4.4 Pillar 4: alignment

Unique to humans are moral and ethical orientations. Individuals and groups have various approaches to what is considered good or not good (morals), and the appropriate conduct in certain contexts (ethics), based on how things are valued, such as the environment (Brennan and Lo, 2016). Though environmental ethics is a major area of philosophy, moral and ethical alignments are less often explicitly considered for stakeholders, yet they harbour the hidden personal drivers of action that produce or restrict transformation. Again drawing on Barad (2010), the relationality of system components means that stakeholders influence and are thus at least implicitly responsible for the things they interact with. Thus, an inherent ethical and moral dimension to stakeholder actions (expressions of agency) in the way a stakeholder impacts other stakeholders, groups, environments, or processes must be accounted for.

Stakeholder characterisation for sustainability transformation therefore requires these moral and ethical orientations to be recognised in some practical way. For this we draw inspiration from the Dungeons & Dragons (D&D) role playing game (RPG) character categorisation system (Gygax and Arneson, 1997). In D&D, players are represented by avatars with combinations of a moral and an ethical alignment. Ewell et al. (2016) found players’ pre-existing D&D moral and ethical alignments usually corresponded to their game avatar. Moral alignments within the game environment are ‘good’, ‘neutral’, and ‘evil’; while ethical alignments are either ‘lawful’, ‘chaotic’, and ‘neutral’^9^ . An avatar described as *lawful good* for example, behaves within accepted laws and rules and is more selflessly helpful to others. *Chaotic good* individuals might have the same inclinations toward good moral behaviour in pursuit of what is right and just but would not respect laws and norms that may impede this. At the other extreme, a *chaotic evil* character would be anti-social, pathologically self-interested, and not beholden to any rule or norm. As such, these archetypes inspire a useful heuristic device for categorising stakeholders along moral and ethical axes, to mapping expressions of their agency within a system, and help to gamify stakeholders in scenario exercises or other methods. The D&D moral-ethical system is found to analytically fit the ‘agreeableness’ trait in the established psychological ‘Big 5′ traits personality test (Ewell et al., 2016).

We recognise the use of terms such as *good* and *evil* may be considered highly subjective and static (even pejorative) labels in relation to stakeholders, especially for researchers wishing to engage them in good-faith. We instead suggest a less categorical or biased interpretation of these alignment's different dimensions to recognise that a stakeholder may occupy more than one moral-ethical position and a range of rationales for their actions without diabolical intent. For example, an NGO that engages in advocacy, research, and direct action might occupy multiple lawful, neutral, and chaotic positions while also holding to be morally righteous and seek to obstruct some groups and benefit others. Thus, we propose a more usable and politically neutral format for stakeholder researchers that is less stark in its assertions about ethics and morality and focussed on the research question. We offer a condensed list of (phosphorus) sustainability-specific alignments that can be adapted to other challenges (Table 4). The alignment of the stakeholder with (phosphorus) sustainability is reflected in the active or coincidental stance for or against (phosphorus) sustainability.

**Table 4:** Alignment with phosphorus sustainability.

Alignment	Definitions	Phosphorus examples
Actively pro-P sustainability *‘lawful and chaotic good’*	Actively working for phosphorus sustainability.	A sustainable phosphorus stakeholder platform.
Coincidentally pro-P sustainability *‘neutral good’*	Indirectly or passively aligned with phosphorus sustainability.	A broader environmental management authority aimed sustainable landscapes or waterways but without a specific term of reference for phosphorus.
Ambivalent *‘neutral’*	No direct or indirect alignment for or against phosphorus sustainability.	A business for farm that may take on a pro or anti-phosphorus practice or technology depending on the subjective benefits or costs.
Actively anti-P sustainability *‘lawful and chaotic evil’*	Actively works against phosphorus sustainability.	A phosphate rock importer dependent on the status quo.
Coincidentally anti-P sustainability *‘neutral evil’*	Indirectly or passively contrary to phosphorus sustainability.	A political party that dismisses environmental concerns, but without a specific reference to phosphorus.

To this point, we have covered agency, role, power and influence, and alignment in reference to an issue. For systems change or transformation, especially in a sustainability context, we must understand stakeholder potential for change.

### 4.5 Pillar 5: transformational readiness

Therefore, the final dimension of our stakeholder characterisation approach is an assessment of their ‘readiness for change’ (Fixsen et al., 2009), that is, how amenable a stakeholder is to transformation, in terms of outlook, role, or functioning. Similarly, recent empirical research on community resilience suggests that the flexible incorporation of different perspectives promotes social cohesion and experimentation with novel transformational practices (Gram-Hanssen, 2019). A key element of transformational readiness for stakeholders is therefore their degree of awareness of the workings of the current system and a future-orientated awareness of alternatives or transition pathways (Carmi and Arnon, 2014; Goodman et al., 2017) – itself a reflection of elements within the other pillars. Accounting for the transformational readiness of the stakeholder assemblage therefore allows us to develop a sense of transformational potential of the system (Marsden et al., 2018).

For our stakeholder-level characterisation, we also attempt to gauge aspects of their future orientation or transformational readiness in order to establish who may represent current or latent ‘leveragers’ or catalysts within the system. Table 5 reflects the provisional characteristics of transformational readiness a stakeholder may possess. *Consciousness* reflects the fundamental awareness of the stakeholder about the phosphorus challenge, sustainability, or other issues or challenges that may directly or indirectly connect to phosphorus. *Openness* describes their attitude to transformation or change-making; for example, more risk-averse or open-minded. For example, within the wastewater sector in European Union (EU), some wastewater companies have a conservative, law-abiding, risk averse culture, which will result in reinforcing the status quo (e.g. treatment processes that simply minimise phosphorus discharges to water in accordance with the EU Water Framework Directive). However, the more innovative open-minded wastewater companies are actively exploring opportunities to recover phosphorus as a marketable product for resale as a renewable fertiliser, along with recovering energy and other valuable resources for the future. *Embodying momentum* is a means of capturing the inertia of a stakeholder’s function in a given system, whether this is for or against change. *Acting* describes whether a stakeholder is acting for change. Our methodological question therefore asks who are the stakeholders that inhabit the business-as-usual, transitional, and transformational spaces in a system (e.g. Sharpe et al., 2016).

**Table 5:** Stakeholder transformational potential and phosphorus examples.

Transformational readiness	Definitions	Phosphorus examples
Consciousness	Awareness about issues that (in)directly connect to phosphorus.	Members of a sustainable phosphorus platform.
Openness	Attitude to change, receptiveness to alternative or new ideas and practices around phosphorus use, recovery, or management.	A water company or farmer willing to try new technologies or practices.
Embodying Momentum	Inertia for transformation – level of commitment to pursuing major shift in how phosphorus is used or managed.	A farmer or company already significantly changing their phosphorus practice past a point of no return, or an agronomist trained to advise on conventional fertilisers.
Acting	A stakeholder presently acting for a sustainable way of managing phosphorus.	A sustainable phosphorus platform member.

## 5 Discussion

Stakeholder analysis in sustainability and transformation has typically involved people or groups with an immediate or direct interest in the issue in question and may not examine closely more nuanced attributes of stakeholders beyond this point because of the inherent difficulties of considering actors and agents in a future system context. The now-common approach to studying environmental problems through complex system lenses (e.g. as social-ecological systems) implies carefully considering the complexity of the system features. For stakeholders, as actors, this means closely examining their individual characteristics, their system-level fit, relative alignment around an issue, and dynamic qualities. Moreover, where a more deliberate consideration of stakeholders is undertaken, the resulting selection may reflect the relative access the research has to them, as some researchers have more privileged access than others. To advance the ability to more deeply understand stakeholders that reflects complexity, even for researchers without direct access, we have developed a five-pillar approach, using the example of phosphorus sustainability.

### 5.1 Applications

We see several major conceptual and practical applications of our approach. First, providing descriptions of stakeholders according to any of the categorisations within the five pillars that may fit, allows for a granular and critical assessment of particular stakeholder attributes (e.g. individuals, groups, or organisational actors with roles in a system) and the wider system. This may be useful for in depth qualitative case studies of specific organisations operating in a larger context, and allow for tailored interviews, survey, or workshop instruments, which could be used longitudinally to capture dynamics. Second, the approach allows for a critical assessment of stakeholders that can occur based on secondary sources (e.g. organisational websites, publicly available policy documents) in instances where stakeholders are inaccessible or in advance of primary data collection where the initial assessment of the stakeholder can be adjusted following closer engagement. Electronic survey software, spreadsheets, printed paper or field notes, or similar means can be used to create editable files for multiple stakeholders in advance of or during fieldwork. Further, drawing on the Dungeons & Dragons inspiration mentioned earlier, our approach is potentially amenable for gamification using either specific or archetypical forms of stakeholders derived from empirical data on real-world stakeholders. For example, rapid advancement of AI coupled to agent-based systems analysis could soon become part of scenario testing in complex systems research where stakeholders may be viewed as avatars. Additionally, this approach, recognising the dynamism of actors, may also allow us to better understand how the system might shape some stakeholders as much as other stakeholders shape the system, as these structure-agency dynamics progress over time.

As a final point, we stress the need for researchers to reflect on the normative *intent* in their research and context when employing this tool. The approach we describe is inherently context-dependent and relates to the interaction of the researcher with both stakeholders and the system. Evidence drawn from primary or secondary sources allows researchers to make judgements about the stakeholder classifications, inclusions and exclusions, and the relationship between stakeholders and the system in which they exist. For example, in the phosphorus and food system examples, three of the normative dimensions to consider include:

1 Aim of the research, to assess system-wide conditions (i.e. unsustainable) for developing phosphorus and food system sustainability, which in turn stem from the normative intent of established environmental and food rights embodied in initiatives like the Sustainable Development Goals;
2 Roles of the stakeholders (i.e. complex and variable) in relation to research aim;
3 Pathway of the system (i.e. mal/adaptive or un/sustainable) in which both researchers and stakeholders are embedded when taken as a whole, in relation to research aim and stakeholder roles.

Considering the subjectivity of the researchers in this way is thus key to facilitating both research transparency and impact, especially if the research is intended to step beyond passive observation to enable change or transformation away from some perceived undesirable or unsustainable state (Fazey et al., 2018b).

## 6 Conclusion

Drawing on the example of the case of phosphorus sustainability, we have proposed five pillars for stakeholder analyses in sustainability transformations. The first pillar conceives of stakeholders as dynamic agents within the system who can change their behaviours and outlooks over time, rather than as static functionaries performing a defined role. The second pillar relates to the system-level role of the stakeholder, and the forms they play or could play in the overall functioning of the system. The third pillar assesses the power and influence stakeholders may wield or gain in a transformed system. The fourth pillar captures interpretations of the moral and ethical alignment relative to a given issue or topic (phosphorus, in our example) that can be broken down into the relative intentionality of the qualities of for, neutral, or against a given idea or practice. The fifth pillar is the transformational readiness of a stakeholder. This pillar is particularly important for sustainability challenges as it is increasingly recognised that we must radically transform worldviews and practices if it is to avoid the deeply adverse consequences of anthropogenic environmental degradation.

Our intention is to encourage reflection rather than prescription. Our view is that stakeholders, as people or organisations (or possibly nonhuman things), are complex actors within a complex social-ecological system facing significant challenges. Finding ways to clearly recognise and meaningfully mobilise the complexity of individual stakeholders as much as the overall system, is a sustainability challenge in its own right, and we hope that other researchers see our work as a stepping-stone to further innovation in stakeholder analysis.

Finally, we stress that this approach is propositional and broad enough to be applied to other cases or any context involving stakeholders. We therefore invite readers to experiment with and critique the ideas we present in this paper.

## Declaration of Competing Interest

The authors declare that they have no known competing financial interests or personal relationships that could have appeared to influence the work reported in this paper.

## Footnotes

1 The definition provided by Freeman (2010, p. 49) refers to “those groups who can affect or are affected by the achievement of an organization's purpose” not a problem. We substitute “organization’s purpose” with “sustainability problem”, which we see as involving multiple stakeholder organisations with differing purposes but key to addressing the issue at hand.

2 An avatar is defined as an icon or figure representing a particular person in a video game, Internet forum, table top role playing, and similar venues in entertainment or learning contexts (Schrader, 2019).

3 https://www.phosphorusplatform.eu/

4 Reference is made to the phosphorus problem in the remainder of this paper; however, the approach we present may be applied to other sustainability contexts.

5 SPA is a forum set-up to advocate for “sustainable use, recovery, and recycling of phosphorus in the food system” (Sustainable Phosphorus Alliance, n.d.)

6 For future work, we also see potential to explore nonhuman features as stakeholders, such as those comprising an ecosystem, that influence each other in complex ways and shape action. For example, the phosphorus is an element that shapes human agri-food and water practices, and as such it could be read as a stakeholder.

7 The question of what is agency and how it works permeates social theory (Archer, 2000; Giddens, 1984; Latour, 2005) but a full review is well beyond the scope of this paper.

8 Morocco occupies Western Sahara—the last colony of Africa—and the vast phosphate rock reserves contained within the territory. The occupation (including alleged widespread human rights abuses), and Morocco’s trade of, and profit from Western Sahara’s phosphate is condemned by the UN and not recognised by other African nation. Many countries, banks and large pension funds (notably Scandinavian), have now divested from companies importing Moroccan phosphates sources from Western Saharan siting ethical and corporate social responsibility motivations (WSRW 2015 & Cordell et al., 2015).

9 Various revisions of the Dungeons & Dragons game use one or two axes character alignments of different points. We drew on the nine-point, two-axes model that inspired Ewell et al. (2016) and features in recent player-tested revisions to the game (Merls and Crawford 2015) for our heuristic: http://media.wizards.com/2016/downloads/DND/PlayerBasicRulesV03.pdf
